# Depressive Symptoms in the Elderly—An Early Symptom of Dementia? A Systematic Review

**DOI:** 10.3389/fphar.2020.00034

**Published:** 2020-02-07

**Authors:** Wietse Wiels, Chris Baeken, Sebastiaan Engelborghs

**Affiliations:** ^1^Department of Neurology, Universitair Ziekenhuis Brussel, Brussels, Belgium; ^2^Center for Neurosciences (C4N), Vrije Universiteit Brussel, Brussels, Belgium; ^3^Department of Psychiatry, Universitair Ziekenhuis Brussel, Brussels, Belgium; ^4^Ghent Experimental Psychiatry (GHEP) Lab, Department of Psychiatry and Medical Psychology, Ghent University Hospital, Ghent University, Ghent, Belgium; ^5^Department of Electrical Engineering, Eindhoven University of Technology, Eindhoven, Netherlands; ^6^Department of Biomedical Sciences and Institute Born-Bunge, University of Antwerp, Antwerp, Belgium

**Keywords:** depression, dementia, cognitive decline, Alzheimer, aging, biomarkers

## Abstract

**Background:**

Depression and dementia are common incapacitating diseases in old age. The exact nature of the relationship between these conditions remains unclear, and multiple explanations have been suggested: depressive symptoms may be a risk factor for, a prodromal symptom of, or a coincidental finding in dementia. They may even be unrelated or only connected through common risk factors. Multiple studies so far have provided conflicting results.

**Objectives:**

To determine whether a systematic literature review can clarify the nature of the relation between depressive symptoms and dementia.

**Methods:**

Using the Patient/Problem/Population, Intervention, Comparator, Outcome or PICO paradigm, a known framework for framing healthcare and evidence questions, we formulated the question “whether depressive symptoms in cognitively intact older adults are associated with a diagnosis of dementia later in life.” We performed a systematic literature review of MEDLINE and PsycINFO in November 2018, looking for prospective cohort studies examining the aforementioned question.

**Results:**

We critically analyzed and listed 31 relevant papers out of 1,656 and grouped them according to the main hypothesis they support: depressive symptoms as a risk factor, not a risk factor, a prodromal symptom, both, or some specific other hypothesis. All but three studies used clinical diagnostic criteria for dementia alone (i.e., no biomarkers or autopsy confirmation). Several studies contain solid arguments for the hypotheses they support, yet they do not formally contradict other findings or suggested explanations and are heterogeneous.

**Conclusions:**

The exact nature of the relationship between depressive symptoms and dementia in the elderly remains inconclusive, with multiple studies supporting both the risk factor and prodromal hypotheses. Some provide arguments for common risk factors. It seems unlikely that there is no connection at all. We conclude that at least in a significant part of the patients, depressive symptoms and dementia are related. This may be due to common risk factors and/or depressive symptoms being a prodromal symptom of dementia and/or depression being a risk factor for dementia. These causal associations possibly overlap in some patients. Further research is warranted to develop predictive biomarkers and to develop interventions that may attenuate the risk of “conversion” from depressive symptoms to dementia in the elderly.

## Introduction

### Rationale and Objectives

Neuropsychiatric diseases are a leading cause of disability worldwide, with numbers expected to increase dramatically in the coming decades, mainly due to aging populations (Alzheimer’s Disease International, 2013). Possibly the most incapacitating of these illnesses is dementia—causing substantial physical and psychological disability, suffering, dependency, and economic costs for patients, caregivers, and society alike (Livingston et al., 2017). Several potentially disease-modifying drug trials may have failed because a disease like Alzheimer’s (AD) is usually diagnosed clinically after underlying pathological processes have already been going on for years, or even decades (Jack et al., 2010; Jansen et al., 2015; Petersen, 2018). This, in turn, has led to a major interest in possible prodromal or (modifiable) risk factors for the development of dementia (Baumgart et al., 2015). Even though most forms of dementia are currently incurable, it has been hypothesized that a 10% reduction of known risk factors could result in a global decrease of more than one million future cases of dementia (Barnes and Yaffe, 2011).

Depressive symptoms have been linked to dementia. Indeed, clinicians have long acknowledged that depression in the elderly can mimic dementia in a situation known as depressive *pseudodementia* (Alexoupoulos et al., 1993). However, depressive symptoms may also be the first clinical manifestation of incipient dementia. Indeed, behavioral and psychological symptoms, such as depression, are highly prevalent in patients with dementia, leading to overlap in clinical presentations of cognitive impairment in the elderly (Savva et al., 2009). Others have suggested that depression and dementia share common risk factors and thereby frequently occur together without being causally linked themselves (Enache et al., 2011), or that psychological symptoms may occur (merely) as a reaction to incipient decline in patients who are aware of their cognitive disturbances. Another explication uses the “cognitive reserve” paradigm. This idea posits that intercurrent (physical or mental) illness in an already diseased and/or aged brain will lower the threshold for experiencing cognitive problems and therefore cause symptoms of the same pathophysiological process to manifest earlier (Stern et al., 1994). Multiple studies designed to assess risk and causality have provided conflicting results (Bennet and Thomas, 2014).

Depression in the elderly is more often associated with cognitive symptoms as compared to depressive disorders of earlier adulthood (Lam et al., 2014). On the other hand, depression itself may actually cause cognitive decline—conceivably related to certain pathophysiological processes of, for example, frontal and hippocampal atrophy possibly through glutamatergic or steroid-related toxicity (Peavy et al., 2007; Byers and Yaffe, 2011; Taylor et al., 2013). Still other studies have pointed out that even early-life depressive episodes increase the risk of later dementia (Dotson et al., 2010; Simões do Couto et al., 2016). The aforementioned possibilities are, of course, not mutually exclusive and quite possibly overlap in everyday clinical situations.

It is clear that the association between late-life depression and dementia is complex. To shed further light upon this issue, we conducted this systematic literature review. It focuses on the relationship between depressive symptoms that develop late in life and the subsequent development of dementia in general.

We acknowledge that depression (as in major depressive disorder) and depressive symptoms are not interchangeable terms. Identifying significant depressive symptoms, rather than limiting studies to those restricting themselves to clinician-ascertained major depressive episode alone, however, will broaden the scope of this review and include more large-scale epidemiological studies. Clinicians, also, will recognize the importance of depressive (and other neuropsychiatric) symptoms that are not severe enough to lead to a formal syndromal diagnosis. A similar rationale was used to examine dementia in a broad sense. Although AD is the most common and best studied form of dementia, vascular and mixed etiologies will not be excluded from our review as they contribute significantly to the aforementioned epidemiological and clinical problems (Alzheimer’s Disease International, 2013; Livingston et al., 2017). Assessing the studies obtained will help identify the gaps in our knowledge that may guide specific future research.

## Methods

### Research Question

To define our research question, we used the Patient/Problem/Population, Intervention, Comparator, Outcome or PICO paradigm—a well-acknowledged framework for framing healthcare and evidence questions, as well as a useful tool to develop concrete questions in complicated and multifactorial issues such as the one we set out to examine. Through a systematic literature review, we studied “whether depressive symptoms (I) in cognitively intact older adults (P) are associated with a diagnosis of dementia later in life (O), diagnosed using validated biomarkers or criteria, as compared to nondepressed matched controls (C).”

### Design and Protocol

Using PRISMA as guidance (Moher et al., 2009), we included human longitudinal, prospective cohort studies reporting on a possible link between depression and depressive symptoms in the elderly (older than 65 years of age) and later development of dementia (not merely cognitive decline in a broader sense) in statistical, and not merely narrative, terms. We did not include case series or other designs to minimize bias, as prospective studies are acknowledged to be less vulnerable to certain forms of bias when ascertaining hazard and risk relationships, especially over longer periods of time. Comparator groups were defined as matched elderly subjects without depressive symptoms. We included memory clinic as well as general community-based population studies of the aforementioned types. No specific length of follow-up was required. There were no restrictions on diagnostic criteria nor rating scales used for detection of depression or dementia, as long as they were clearly defined and respected. We excluded studies ascertaining similar problems in highly specific pathological situations, such as Huntington’s disease, Down syndrome, or prion diseases. No language or publication date restrictions were applied.

Medline and PsycINFO databases were searched in November 2018 using combinations of the following terms we identified through the PICO paradigm: depression (including variant wordings such as “depressive symptoms” in MeSH), dementia, Alzheimer, elderly, incidence, risk, hazard, cohort. We subsequently added search terms containing clinical diagnostic biomarkers such as magnetic resonance imaging (MRI), positron emission tomography (PET), cerebrospinal fluid (CSF), biomarkers, amyloid, tau, and neuropsychological test/examination (see **Supplementary Material Table** for these keywords and combinations used).

We collected and deduplicated references using EndNote software (*Clarivate Analytics*). Titles and abstracts were screened by carefully excluding publications irrelevant to our research question (mainly *in vitro* studies, cross-sectional designs, papers about highly specific other illnesses as mentioned above, case studies…—i.e., publications clearly incompatible with our inclusion criteria and research question). Studies with possibly relevant contents were fully read and considered for inclusion using the aforementioned inclusion and exclusion criteria and preparing to resolve possible conflicts on study inclusion or exclusion (which did not occur) among the three authors by consensus. We further screened the references of these articles for missed relevant publications. All were evaluated for possible objective errors. No studies found through PsycINFO were unlisted in Medline searches. As such, all (n = …) refer to references obtained from Medline. We used the Newcastle–Ottawa Scale (NOS) for cohort studies to assess risk of bias (obtained from ohri.ca/programs/clinical_epidemiology/oxford.asp) in prospective cohort studies.

## Results

**Figure 1:**
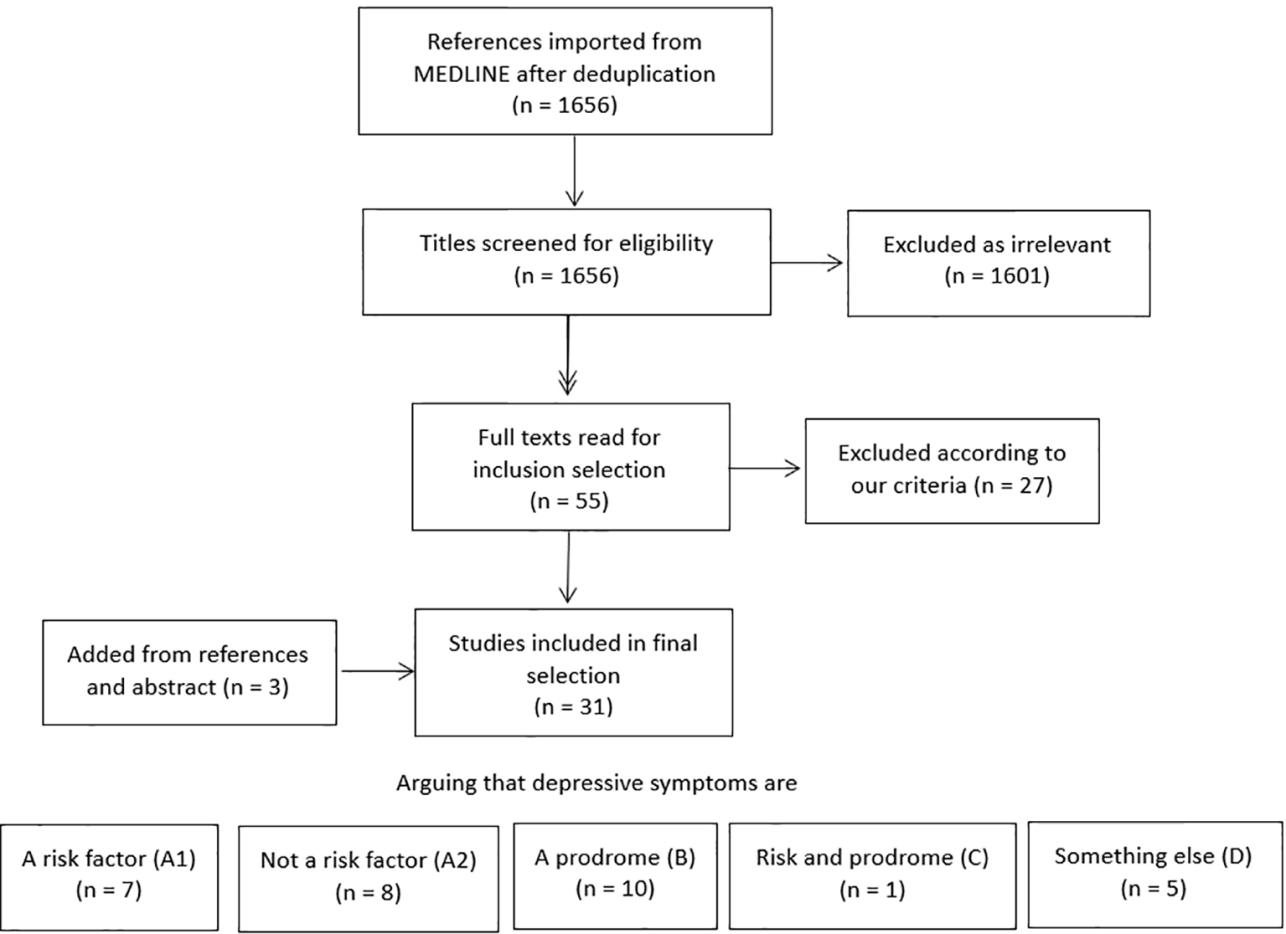
Selection of studies included in this systematic review.

Results are listed in **Figure 1**. Out of 1,656 search results, 1,601 titles and abstracts were excluded as clearly irrelevant. Fifty-five full articles were read and evaluated, of which 27 were excluded according to our predefined inclusion criteria. In our final assessment, 31 studies were included. We briefly mention seven additional publications that looked at cognitive decline *sensu lato* rather than dementia and five studies that included many patients deemed too young as per our cutoff of 65 years. Three papers by Wilson et al. (Wilson et al., 2003; Wilson et al., 2014; Wilson et al., 2016) described similar cohorts and neuropathological data and were merged into one additional reference. One additional study was added through follow-up for publication of an earlier abstract of interest the authors read at a conference (Ezzati et al., 2019). Two other papers were included from paper references. No data were extracted as we considered the obtained papers to be too heterogeneous to perform meta-analysis.

We grouped these references in five categories for review purposes—noting that their main conclusions are not mutually exclusive and many authors, indeed, suggest multiple explanations for their findings. Critical assessment of the papers’ numerical results as well as their authors’ main interpretation thereof in the respective discussion sections was used to classify the references in our different categories, as discussed further in this section. Additional substantiation is provided in the **Supplementary Material**.

- Studies suggesting that depression is a risk factor for dementia A1 (n = 7) and studies suggesting that depression is not a risk factor for dementia or that they are linked without reaching statistical significance A2 (n = 8)
- Studies that suggest that depression is an early symptom or prodrome of dementia (n = 10)
- Studies that suggest that depression is both an early symptom and a risk factor (n = 1)
- Studies demonstrating an association between depression and dementia, without clear conclusions concerning potential causality [n = 3 (one reference for three related papers)]

Results are listed by their aforementioned category in **Tables 1**–**4** (listing A1, A2, B, C&D).

**Table 1:** (Category A1): Studies suggesting that depression is a significant risk factor for dementia.

Study	Cohort	N	Mean age (SD)	% Female	FU	Depression assessment	Diagnostic criteria of cognitive decline	Incidence of dementia	Risk?	Risk adjustments
(Brodaty et al., 2012)	Community of English speaking Australians	799 (480 NC, 319 MCI). 169 drop-outs	78 (4.7)	67.8%	2 years	NPI	Petersen MCI, DSM-IV Dementia	11 from MCI, 3 from NCI, too small for subtyping	OR 3.67 [1.1–12.5]	No difference (age, sex, education, NPI score)
(Burke et al., 2016)	Prospective UDS NACC: 80% White, 13% African, 6% Hispanic	11453 start, 8762 more than 1 visit	71 (10.89)	65.2%	Mean 3 years (1-10)	1) Recent 2) Earlier 3) Current episode (DSM-IV)	AD NINCDS/ADRDA	330 AD. Subgroups not clearly reported	1) HR 2.35 [1.88–2.94] 2) HR 1.35 [1.06–1.73] 3) HR 2.82 [2.21–3.59]	Stronger after correction 1) HR 5.75 [3.28–10.07] 2) 3.20 [1.78–5.73] 3) HR 5.50 [3.09–9.64]
(Devanand et al., 1996)	Community in Manhattan 30% White, 41% Latino 27% African	852 NC at baseline, 478 one or more FU sessions	73 (7.1)	69.4%	1-5, mean 2.54 years (1.12 SD)	HAM-D >10 and ‘mood’ item	DSM-III-R	61 cases, (21% depressed vs 9% non), mostly AD (2 other, 1 PSP)	RR 2.94 [1.7-4.9]	Still significant (RR 2.05) after age, education, Moderate CI
(Ezzati et al., 2019)	Einstein Aging study: Bronx community, 65% white	1219	78 (5.4) 70+	62%	Yearly visits for 4.5 years (3.5 SD) up to 17 years	GDS-15	DSM-IV	132 cases, 111 AD	Per point HR 1.11 [1.03-1.19], not significant before 3 years	Age, sex, race, education, comorbidity and baseline cognition (BIMC)
(Irie et al., 2008)	USA Japanese in Hawaii	1932	76 (3.6)	0%	6.1 years mean	CES-D > 9	DSM-III-R, NINCDS/ADRDA	6.3% (e4), 9.3% (dep) 13.7% (both) 4.2%(neither)	Dementia HR 2.2 [1.3–3.7] AD 2.9 [1.4–5.9]	Classic risk factors and self-reported memory
(Saczynski et al., 2010)	Framingham study: prospective community cohort	949	79 (5)	63.6%	Mean 8, up to 17 years	CES-D > 16	DSM-IV, CDR >1, NINCDS/ADRDA	164 cases (136 AD)	Dementia HR 1.72 [1.04–2.84] AD 1.76 [1.03–3.01]	Age, education, homocysteine, APOE, MCI
(Spira et al., 2012)	Oldest old white (SOF WISE)	302	87 (2) > 85	100%	5 years after baseline	GDS-15 > 6	Petersen MCI, DSM-IV-TR dementia	84 cases 65% of GDS ≥6 & 37% < 6	MOR 3.15, [1.03-9.65]	Risk factors, alcohol, benzodiazepines

Values in square brackets are 95% confidence intervals. p-values are under 0.05 unless otherwise specified. UDS-NACC, Uniform Data Set - National Alzheimer’s Coordinating Centre; NC, Normal cognition; MCI, Mild Cognitive Impairment; SD, Standard Deviation; GDS, Geriatric Depression Scale; HAM-D, Hamilton Depression Rating Scale; NPI, Neuropsychiatric Inventory; DSM, Diagnostic and Statistical Manual of Mental Disorders (American Psychiatric Association); FU, follow-up, NINCDS; National Institute of Neurological and Communication Disorders and Stroke; ADRDA, Alzheimer’s Disease and Related Disorders Association criteria; CES-D, Centre for Epidemiological Studies Depression scale; BIMC, Blessed Information Memory Concentration; CDR, Clinical Dementia Rating Scale; SOF-WISE, Study of Osteoporotic Fractures - Women; Cognitive Impairment Study of Exceptional Aging.

**Table 2:** (Category A2): Studies not suggesting that depression is a significant risk factor for dementia.

Study	Cohort	N	Mean age (SD)	% Female	FU	Depression assessment	Diagnostic criteria of cognitive decline	Incidence of dementia	Risk?	Adjustments
(Becker et al., 2009)	CHS-CS Pittsburgh community	288	78 (3.65)	63%	7.1 years (1–9)	CES-D >10	3MSE, DSM-IV, NINCDS/ADRDA	48, all AD	None found	All kinds of risk factors in CHS study
(Blasko et al., 2010)	VITA – 2 Vienna districts	331 never depressed undemented	76 (0.5)	56.5%	0 – 2.5 – 5 years	DSM-IV, sGDS	NINCDS/ADRDA	33	Serum Ab42, male gender, age were risks	Multivariate logistic regression analyses
(Geerlings et al., 2008)	Rotterdam population Scan Study	486	74 (6.5)	49%	5.9 years (1.5 SD)	Interview-based (early vs. late (60y)) CES-D 16	CAMDEX and standard criteria	33 AD	EARLY history of depression risk factor	MRI volumetrics and WML
(Kim et al., 2010)	Korean community survey	518	72 (5) 65+	54.4%	2.4 years	Korean GDS	Standard criteria	45 (34 AD, 7 VaD, 4 other)	Strong risk of dep + APOE men (cf. Irie)	Vascular risk factors, APOE
(Lindsay et al., 2002)	Prospective Canadian Health & Aging study	4609	Cases 87 Controls 78 (70–100)	58%	5 years after initial visit	DSM-III-R (no symptoms only)!	NINCDS-ADRDA, DSM-III, NINDS-AIDEN	194 AD, 527 MCI and ‘other’ dementias	Age, education, APOE	Reduction by wine, coffee, NSAIDs, exercise,…
(Mossaheb et al., 2012)	VITA – 2 Vienna districts	437 (296 at 60 months) never depressed	76 (0.5)	55%	60 months	DSM-IV-TR questionnaire, HDRS, sGDS	NINCDS-ADRDA	65 (AD)	OR 5.27 [1.62-17.2] for loss of interest only	Risk factors, biochemical parameters, APOE
(Pálsson et al., 1999)	Gothenburg census of 85 year olds	227 healthy and 62 depressed	85 years old at baseline	Not explicitly stated in subgroups	3 years	DSM-III-R, history and records	Not clearly stated, MMSE	50 cases	Only early onset MDD	CT volumetrics
(Vinkers et al., 2004)	Leiden (NL) 85 year olds	500 (298 4^th^ year)	85 years old at baseline	63%	Yearly for 4 years	GDS-15	MMSE, Stroop, LDCT, 12 Word Recall	Not explicitly stated	Correlation but no risk of decline	Mixed models

3MSE, Modified Mini Mental State Examination; WML, White Matter Lesions; AIDEN, Association Internationale pour la Recherche et l’Enseignement en Neurosciences; MDD, Major Depressive Disorder; LDCT, Letter Digit Coding Test.

**Table 3:** (Category B): Studies suggesting that depression is a prodromal symptom of dementia.

Study	Cohort	N	Mean age (SD)	% Female	FU	Depression assessment	Criteria for cognitive decline	Incidence	Risk?	Adjustments
(Almeida et al., 2017)	Health in Men (Western Australia)	4922	77 (3.7) 71-89	0%	8.9 mean, up to 14 years	sGDS-15, history, health record	Healthcare records coding	903 cases (18%)	aSHR: Ever 1.3 [1.2-1.7] Past 1.3 [1.0-1.6] Current 1.5 [1.2-2.0]	Antidepressant use, stroke, risk factors. ONLY in the first 5 years
(Chen et al., 1999)	MoVIES cohort USA 97% white	954	65-85	54.6%	8 years with 2 yearly intervals	mCES-D	NINCDS-ADRDA, DSM-III, CERAD	78 (61 AD)	‘Reverse’ risk	Age, sex, education, self-reported cogn.
(Fuhrer et al., 2003)	Aquitaine France community	3777 (1500 at year 8)	75 (7) 65+	58.3%	8 years (1, 3, 5, 8)	CES-D (men 17 women 23)!	NINCDS-ADRDA, DSM-III-R, Hachinski	280 cases (200 AD)	OR men 3.5 [1.9–6.5] Women [1.2 0.7–2.0]	Hypertension in men 50% additional risk
(Gatz et al., 2005)	Manitoba Canada Community	766	75 (6) 65+	61.7%	5 years	CES-D (> 16 and other values)	Standard Criteria	56 (36 AD)	AD OR 2.75 [1.04–7.24] Dementia 2.37 [1.02–5.54)	Not earlier reported depression, not duration of symptoms
(Geerlings et al., 2000)	Two Netherlands cohorts	1911 + 1894	73 (5) and 70 (8)	62.3% and 52.9%	4 years	GMSS and CES-D respectively	“3 point MMSE drop” and criteria	53 AD AMSTEL	aOR >8y educated 5.3 [1.8-15]	Age, gender, education, psychiatric
(Lenoir et al., 2011)	Three French Cities (3C Study)	7989	74 (5) 65+	61%	2 times 2 years	MDE-MINI (DSM IV), CES-D	NINCDS-ADRDA, DSM-IV, Hachinski, AIREN	180 AD, 24 Vasc Dem, 29 mixed, 43 various	Dementia HR 1.5 [1.2–2.2] Vascular 4.8 [2.2–10.7	Risk factors and age, MRI WML, not earlier episodes
(Li et al., 2011)	Seattle ACT study	3410	75 (6) 65+	60%	7.1 average, Biennially up to 15 years	CES-D-11, history of past episodes	CASI, DSM-IV, NINCDS-ADRDA	658: 386 AD, 89 Vasc, 113 mix, 70 other	All cause aHR 1.61 [1.29- 2.01]	Age, gender, education, baseline CASI
(Mirza et al., 2016)	Rotterdam community scan study	3325	74.88 (IQR 71–80)	60%	3 times in 10 years	CES-D, HADS-D – 3 trajectories!	MMSE, GMS, CAMDEX, DSM-III-R, NINCDS-ADRDA	434 dementia – 348 AD, 26 Vasc, 60 other	Increasing trajectory dementia HR 1.42 [1·05–1·94]	Age, sex, *APOE*, education, medication, risk factors, cognition
(Palmer et al., 2007)	Stockholm community-based	185 (+47 MCI)	84 (5) 75-95 years	84.9%	3.4 years (0.6 SD)	Comprehensive Psychopathological Rating Scale	DSM-III-R, NINCDS-ADRDA	10 AD 7 dementia in healthy	AD RR 1.9 [1.0-3.6] per mood symptom	Corrected for age, sex, education. Anxiety in MCI
(Verdelho et al., 2013)	LADIS study of WML (clinic finding based)	639	74.1 +- 5y	55%	3 times annually	sGDS	NINCDS-ADRDA/AIREN	34 AD, 54 VD, 2 FTLD	GDS HR 2.4 [1.4 3.99]	Risk factors. Previous depression not significant

aSHR, Adjusted sub-hazard ratio; CERAD, Consortium to Establish a Registry for Alzheimer’s Disease; GMSS, Geriatric Mental State Schedule; MDE-MINI, Major Depressive Disorder part of the Mini International Neuropsychiatric Interview; CASI, Cognitive Abilities Screening Instrument; IQR, Interquartile Range; WML, White Matter Lesions.

**Table 4:** (Categories C & D): Studies suggesting both or neither.

Study	Cohort	N	Mean age (SD)	% Female	FU	Depression assessment	Diagnostic criteria of cognitive decline	Incidence	Risk?	Adjustments
(Ganguli et al., 2006)	MoVIESBlue-collar rural U.S.A	1265	75 (5)	60.8%	Biannually for 12 years	mCES-D	CERAD, DSM-III-R	171 cases of dementia	Multiple interaction models finding no long-term association	Classic risk factors
(Kaup et al., 2016)	HABC Study, mixed USA	2488	74 (2.4)	53%	4 years	CES-D-10 and ‘trajectories’	Records, medication, MMS decline	353 cases of dementia	High and increasing trajectory	Demographic and health factors, cognition
(Luppa et al., 2013)	LEILA 75+ Study	1265	75 years and older, mean 81	73%	Every 1.5 years over 8 years	CES-D	SIDAM - DSM IV	183 cases of dementia	Only for MD in multivariate	Classic risk factors
(Wilson et al., 2003; Wilson et al., 2014; Wilson et al., 2016)	Different prospective cohorts	130/1750/1965	76 (7.5) in 3rd study	73.8%	Differing per cohort/paper	CES-D	DSM-III, NINCDS-ADRDA, Pathological criteria	346 cases of dementia in largest cohort	Cfr. text discussion.	Cfr. text discussion.

SIDAM, Structured Interview for Diagnosis of Dementia of Alzheimer type, Multiinfarct Dementia and Dementia of other Aetiology; MD, Major Depression.

Bias assessment using the NOS did not show any systematic difference in biases between categories, with all studies scoring relatively high on this design quality scale. We therefore conclude this had little influence on our findings. Results and additional comments are available in the **Supplementary Material Table**.

These studies propose that depressive symptoms confer an additional risk of a future development of dementia in cognitively healthy elderly individuals. This view has to be contrasted with the hypothesis that psychological symptoms simply accentuate or temporarily cause cognitive deficits, thereby accounting for that proportion of mild cognitive impairment (MCI) patients who do not “convert” to dementia but rather recover a normal cognitive status (Langa and Levine, 2014). Some studies (Irie et al., 2008; Ezzati et al., 2019) indeed corrected for baseline emotional and cognitive scores to address this possibility. We did not include studies evaluating the link between early-life (onset before 65 years of age) depression and later dementia, even though this could be of interest to our research question; indeed, other review efforts appear to suggest this link (Ownby et al., 2006). Interestingly, several studies, including the largest one (Burke et al., 2016) in this category, suggest that dementia risk in people with depressive symptoms increases further when corrected for classically biasing factors such as age, education, or socioeconomic status, thus providing an argument against the position that depressive symptoms and dementia are linked solely through common risk factors and justifying classification in category A1. This relation becomes even stronger when associated with certain *APOE* genotypes, a well-known genetic risk factor for AD—a finding also reported by smaller samples (Irie et al., 2008; Kim et al., 2010).

Considering some of the proposed pathophysiological mechanisms (chronic inflammation, glucocorticoid toxicity, neuronal energetic dysregulation, etc.), a true biological risk relationship would imply that more severe and/or long-standing depression increases one’s chance of cognitive deterioration due to a neurodegenerative or cerebrovascular brain disease. Although some reports (Wilson et al., 2003; Gatz et al., 2005; Saczynski et al., 2010; Langa and Levine, 2014) have shown such a link, most studies use depressive symptoms as a binary value (depressed–not depressed) and/or contain too many individuals with mild depressive symptoms to really establish a “dose–response” relationship of this kind. Furthermore, most studies did not evaluate these proposed mechanisms (e.g., through blood or CSF analysis, functional or advanced imaging). It is etiologically difficult to disentangle depression being a pure risk factor or an actual prodromal symptom of dementia, especially during the 2- or 3-year follow-up of most studies. However, some studies followed patients for up to 17 years and reported similar findings (Saczynski et al., 2010). One strong argument for the true risk hypothesis would be clinical trials indicating that successful treatment of major depressive episodes lowers the incidence of (solidly diagnosed) degenerative dementia. This is a controversial question, with multiple studies of different (mainly pharmacological) treatments providing conflicting results (Lee et al., 2016; Jacob et al., 2017; Almeida et al., 2017; Chu et al., 2018; Brauer et al., 2019). We further note that the successful treatment of clinical depression is a challenge in itself, as only one in three patients respond to first-line treatments and as many remain treatment resistant after multiple treatments, a situation that may be even more frequent in the elderly (Rush et al., 2006). Interestingly, some authors have even suggested that anti-amyloid therapies may have a role to play in treating late-life depressive syndromes (Mahgoub and Alexopoulos, 2016). Nevertheless, the studies we examined here fail to provide us with any additional evidence of this sort.

These studies did not find an increased risk of dementia in case of late-life depressive symptoms, as opposed to the first group of studies. The authors’ point is generally based upon an absence of significant findings or results that lose statistical significance after correction for certain biasing factors. Some, however, do establish a risk relationship in specific situations, e.g., in combination with sex or *APOE* genotype (Lindsay et al., 2002; Kim et al., 2010). Others report an association with depressive episodes earlier in life as opposed to current symptoms (Pálsson et al., 1999; Geerlings et al., 2008). Several studied rather small populations, and their relatively low numbers of incident dementia cases may in itself account for a negative result (Geerlings et al., 2008; Becker et al., 2009; Blasko et al., 2010; Kim et al., 2010). This may also be the case for wide confidence intervals [e.g., in Geerlings et al. (2008), 0.82–6.69]. Some of these did not find any significant risk factors for dementia, apart from increasing age, which stands in apparent conflict with what is generally accepted in dementia research (Baumgart et al., 2015; Livingston et al., 2017) and quite possibly suggests insufficient power. We note that one study (Kim et al., 2010) reported an increased risk of dementia only for depressive males carrying an *APOE* ϵ4 allele. Lindsay et al. (Lindsay et al., 2002) looked only at syndromal (i.e., clinician-diagnosed) depression, as opposed to most of the other studies that utilized (self-reported) symptom scores. Two studies (41 and 44) report on the same cohort. The population studied in (Geerlings et al., 2008) was analyzed again in (Mirza et al., 2016), which is category B and supports the prodrome hypothesis (**Table 3**). Most population sizes in **Table 2** are smaller than those in other groups. In combination with other factors, as mentioned above, we conclude that several of these studies were probably underpowered to detect a connection and do not formally nor decisively contradict the notion that depressive symptoms and dementia are connected.

Considering that both neuropsychiatric illnesses may manifest themselves epidemiologically as hazard increasing, it can be difficult to distinguish between a causal risk factor and prodromal symptom in the years leading up to a diagnosis of dementia—as discussed above. Nevertheless, the authors in this category found support in their findings for the latter hypothesis. Several arguments in favor of this position are advanced by the authors of these studies. These include an increasing symptom burden over time (Mirza et al., 2016) and no effect of (the length of) episodes occurring earlier in life as opposed to current and recent symptoms (Fuhrer et al., 2003; Li et al., 2011). Others note a clear temporal relationship of both illnesses around diagnosis (Lenoir et al., 2011; Almeida et al., 2017) or a synergistic effect with white matter pathology (Verdelho et al., 2013). Concerning this last element especially, it is posited that in some proportion of elderly patients, depressive symptoms actually represent the organic effect of ongoing pathological processes (cerebrovascular and/or neurodegenerative) on affect-modulating networks in the brain (Thomas et al., 2002).

One must also consider here the possibility that depressive symptoms could be a reaction to patients’ awareness of cognitive decline and thereby frequently manifest during prodromal and early stages of dementia. Several authors, however, reported no clear effect of baseline cognition and emotional symptoms to later dementia (Chen et al., 1999; Geerlings et al., 2000; Fuhrer et al., 2003; Irie et al., 2008; Li et al., 2011; Mirza et al., 2016; Ezzati et al., 2019), providing a solid argument against this position. Furthermore, other studies have reported that no clear or ubiquitously negative reaction to dementia in recently diagnosed subjects can be demonstrated (Carpenter et al., 2008). We therefore hypothesize that, even though this may occur in everyday clinical situations (e.g., someone worrying about future cognitive decline, possibly due to contact with the dementing illness of a relative), a negative affective response to noticing one’s own decline alone cannot explain the association between depressive symptoms and dementia in all patients. Future and ongoing studies like the ABIDE project (van Maurik et al., 2019) will be of help to shed further light upon this association.

These studies propose multiple explanations or more complex associations between depression and dementia. Ganguli et al. (2006) (studying the same cohort as Chen et al. (1999) in category B, **Table 3**) hypothesized that, while depressive symptoms are indeed cross-sectionally associated with cognitive symptoms, they were unrelated to later cognitive decline, while noting that increasing cognitive symptoms associated with depression likely represent incipient dementia. Kaup et al. (Kaup et al., 2016) described that increasing severity of depressive symptoms on repeated assessments, rather than a one-time scoring, was associated with increased risk of dementia, thereby lending support to both risk factor and prodromal hypotheses. Luppa et al. (2013), through multiple interaction models, show that only major depressive episodes (i.e., depression in the strict sense) seem to increase risk of dementia as opposed to milder depressive symptoms whose effect disappears in multivariate analyses.

Wilson et al. (2003; 2014; 2016) have published three interesting studies. Their 2003 paper showed that, in a group of 130 elderly religious order members, each increase in depressive symptomatology increased the risk of being diagnosed with AD. However, this did not seem to correlate well with the burden of AD neuropathology at autopsy, leading the authors to conclude that some other mechanism must drive the association between depression and dementia. They confirmed this finding in an expanded cohort of 1,750 people, 600 of whom underwent neuropathological examination, and again in a third paper. They found no support for depression being merely a psychological reaction to incipient cognitive decline but confirmed the association between depressive symptomatology and later dementia. They hypothesized that some mechanism, independent of the postmortem hallmarks of AD (i.e., plaques and tangles), must drive the association between depression and cognitive decline. Exactly what drives this intriguing finding remains to be elucidated in future studies. These should include newer biomarkers, as guided by fundamental scientific insights.

An amyloid-PET-based study has yielded similar results (i.e., showing no clear link with amyloid pathology) when evaluating hippocampal atrophy in a cohort of depressed people and matched controls (De Winter et al., 2017), even though others have pointed out that amyloid-positive individuals do have a tendency to progressively manifest more neuropsychiatric symptoms (Harrington et al., 2017; Donovan et al., 2018). It is clear that further research efforts could and should use the newer antemortem diagnostic techniques (e.g., LP and/or PET) to add to these findings.

## Discussion

Our systematic literature review yielded no conclusive arguments in support of or contradicting the exact nature of the etiological relationship between depression and dementia. Multiple studies, however, contain convincing arguments for the respective position on this topic (A1, A2, B, C, D) that their authors defend. Some issues raised in the *Results* section of category A2 (“not a risk factor”), however, cast doubt on the power several of these studies to detect connections (or their absence). We deem it unlikely that there should be no connection at all, or that everything can be explained by subjects’ emotional responses to incipient decline. We therefore conclude that depressive symptoms may be both a risk factor for and a prodromal symptom to dementia. They may sometimes be coincidental and/or stem from shared risk factors in the elderly. Exactly what mechanism(s) drive(s) this pathophysiological association remain(s) unclear and could not be elucidated by this systematic review. Limitations of our effort in this sense will be discussed in the next section of this article.

Multiple studies were excluded from our main results as they examined the relationship between depressive symptoms and cognitive decline, variously defined (Dufouil et al., 1996; Bassuk et al., 1998; Yaffe et al., 1999; Wilson et al., 2004; Barnes et al., 2006; Geda et al., 2014; Ritchie et al., 2016). Due to this heterogeneous construct (rather than the binary dementia–no dementia), these studies did not answer our research question. They remain, however, certainly of interest of the broader research area we considered in the *Introduction* section. Since these studies contain useful information for investigators looking into the cognitive effects of depression, they are listed here for further reading. Five of them report an increased risk of cognitive decline among depressed elders (Yaffe et al., 1999; Wilson et al., 2004; Barnes et al., 2006; Geda et al., 2014; Ritchie et al., 2016), whereas two of them do not (Dufouil et al., 1996; Bassuk et al., 1998).

As we wanted to study the interaction between late-life depression and dementia, studies including a large proportion of subjects younger than 65 years of age were excluded. Indeed, dementia is rare in this age group, and early- versus late-onset depression may differ clinically (Hall and Reynolds-Iii, 2014). As mentioned before (Ownby et al., 2006), some of these studies suggest that [severe (Simões do Couto et al., 2016)] early-life depressive episodes appear to have an “additive” effect on dementia risk, supporting depression as a causal factor or related to the same underlying pathology, such as vascular disease (Van Uden et al., 2016). Dal Forno et al. (2015) and Köhler et al. (2011) reported similar findings, albeit in older populations. These findings may be relevant to our review question and often imply a causal role for depression and depressive symptoms, much like the studies in Table 1. Even though the studies mentioned cannot be included in our systematic review, given our inclusion criteria, we briefly refer to these studies here (Dotson et al., 2010; Köhler et al., 2011; Dal Forno et al., 2015; Simões do Couto et al., 2016; Van Uden et al., 2016), as most do appear to support a link between depressive symptoms and dementia.

Neuropsychiatric symptoms in the elderly are common, pervasive, and incapacitating. In the search for validated biomarkers of (later) dementia, simple and cheap interventions, such as a structured mental health assessment or a quick screening tool for depressive symptoms, may complement expensive and/or invasive tools like imaging and laboratory tests in determining individual patients’ risk profile. Hopefully, these can in turn lead to tailored risk mitigation strategies for individuals at risk that can be implemented on a large scale. Future research should aim at identifying novel techniques that are able to identify those depressed elders at high risk for “conversion” to dementia. There may be a role to play in unraveling this connection for certain issues raised in the papers we discussed. The effects of gender, genetics, cerebrovascular disease and inflammation, upon the interaction between depressive symptoms and (certain types of) dementia should be studied further using a combination of large datasets and modern technology. We believe that these areas of study may yield clues to understand the actual pathophysiological mechanisms driving the association between mood symptoms and cognitive decline and in turn guide future trials.

We hypothesize that newer diagnostic techniques—*in vivo* biomarkers through CSF analysis, targeted molecular imaging through positron emission tomography techniques, advanced (magnetic resonance) imaging analysis—unavailable during many of the prospective study periods of the cohort studies we cited—may facilitate in this effort in overcoming the shortcomings of existing studies. The studies we included are mainly based on cohorts from years or even decades ago, when much fewer (para) clinical diagnostic tools were available. By increasing diagnostic accuracy and concordance with pathological diagnoses, disease-specific mechanisms may be identified more easily as compared to the more heterogeneous cohorts described above. We theorize that these techniques may facilitate an early identification of those depressed elders at an especially high risk of developing degenerative dementia. Second, future trials should examine whether and which treatments in the depressive elderly—with or without evidence of preclinical or prodromal neurodegeneration—may mitigate their risk of later dementia.

## Limitations and Heterogeneity

### -Of the Studies Included

It is technically and ethically impossible to conduct a randomized, controlled trial to study the association between depression and dementia. Therefore, conclusions are based on observational studies. This means that the highest levels of evidence quality are not met. This is especially relevant when taking heterogeneity across studies into account in conducting systematic reviews and meta-analyses. We note that most studies are of similar quality when assessed using the NOS (see **Supplementary Material**). We therefore deem it unlikely that specific types of systematic bias in study design (apart from some concerns raised over the “negative” studies in Category A2) have influenced the categorization of studies and/or our general conclusions.

### Related to the Diagnosis of Dementia

Dementia diagnoses were based on Diagnostic and Statistical Manual of Mental Disorders (DSM) (be it III or IV, revised or not) criteria in all but two studies (Kaup et al., 2016; Almeida et al., 2017), which relied on healthcare records and coding, prescription of cholinesterase inhibitors, and other “indirect” signs of a dementia diagnosis in their cohorts. AD diagnosis—if evaluated separately—was similarly based on National Institute of Neurological and Communicative Disorders and Stroke and the Alzheimer’s Disease and Related Disorders Association (NINCDS-ADRDA) criteria in most studies. Of note, almost no studies included additional “objective” biomarkers of AD or other dementias, which added to the heterogeneity of the investigated populations. The relatively low etiological diagnostic accuracy of non-biomarker-based clinical diagnostic criteria alone does not help this issue (Elahi and Miller, 2017). A minority of studies employed DNA analysis—mainly for *APOE* genotyping (Lindsay et al., 2002; Irie et al., 2008; Kim et al., 2010; Saczynski et al., 2010; Mossaheb et al., 2012; Mirza et al., 2016). While most criteria for “dementia” as such are quite clear, there are of course multiple causes for this clinical syndrome, and several studies do not formally differentiate between AD and other causes of dementia, very probably contributing to less clear results and precluding conclusions about specific types of dementia and their possible association with depressive symptoms. These issues may have influenced study findings and the conclusions of this systematic review.

### Depression Measures

It is of interest to researchers and clinicians to differentiate between several elements of the depressive syndrome (anhedonia, low positive affect, motivational symptoms, vegetative or melancholic symptoms,…) to elucidate the role they have in causing clinical overlap between multiple disease states in the elderly: medical illness and pharmacological effects, major depression, apathy, incipient dementia, etc. (Hall and Reynolds-Iii, 2014). Rating scales differ in their focus on/attributing points to these elements of the depressive syndrome.

Fourteen studies used the Centre for Epidemiological Studies–Depression (CES-D) self-rating scale for assessing depressive symptoms at a given moment. Nevertheless, even they differ in the cutoff values used (e.g., 16, 11, 10, or 9 points); two studies use a modified scale, some use different cutoff values for men and women, while others did not. No less than nine other scales and evaluations were used, with or without a “regular” clinical psychiatric assessment of depression (generally based on DSM criteria). Ascertaining past episodes of depression was even harder due to recall bias and a general lack of recognition of multiple depressive symptoms and episodes in the general population (Patten, 2003). Although some of the included studies examined specific symptom trajectories of depression, most use a one-time screening tool inquiring about symptoms in the last few days or weeks, which is probably inadequate to distinguish true depression from subsyndromal depressive symptoms. This limits strong conclusions about what kind and/or severity of depressive symptoms are specifically related to future dementia and should, therefore, be actively identified and/or treated by clinicians.

### Populations

Studied populations were relatively heterogeneous across cohorts. Participants’ age was similar in most studies, with some studies focusing on the oldest old (Pálsson et al., 1999; Vinkers et al., 2004; Palmer et al., 2007). While a general female preponderance in studies of the elderly is to be expected and was indeed seen in most studies, some (29 and 47) looked at men only, with one (Spira et al., 2012) having an all-women cohort. Most (if not all) of these studies were conducted in the industrialized world. They do, however, contain populations from different continents and multiple ethnic backgrounds in rural as well as urban cohorts. Whether study findings can be extrapolated on a cross-cultural or global scale remains an open question.

### Risk Analysis and Prospective Studies

While almost all studies corrected their results (concerning risk of developing dementia) for potentially confounding variables, almost none used an exactly identical list of confounders. Given that depressive symptoms and dementia are both common and have overlapping risk factors (e.g., socioeconomic status), this lack of uniformity across studies further hampers the disentanglement of causal relationships.

As in all epidemiological studies, and especially those in old age, attrition bias (due to loss of follow-up, intercurrent illness, or death) is significant. Furthermore, multiple authors have suggested that dropped-out participants are more likely to suffer from depression and/or dementia (Brayne et al., 1999), possibly attenuating the risk found in study “survivors.” Moreover, several authors have pointed out that people with depressive symptoms have more comorbid medical illnesses and die earlier (which is also true for people with dementia) (Almeida et al., 2010). We hypothesize that this attrition bias may underestimate some risk relationships and could produce false-negative results.

### -Limitations of This Review

As can be expected from examining the complex association between depressive symptoms and dementia, major methodological differences exist between studies. There are profound and significant differences concerning the populations studied, diagnostic evaluations used, follow-up frequency and duration, corrections for bias, etc. These inconsistencies add to the difficulty of answering our research question (i.e., depression being a cause, effect, both, neither,… of dementia) that is in itself challenging to answer using prospective studies. Grouping studies and data that are this heterogeneous are a major limitation of the existing data. This also complicates hypothetical statistical analyses of data extracted from these studies.

We do not provide here a complete list of excluded studies, did not do the initial searches in duplicate, and cannot exclude any human errors in selection. Since the link between depression and dementia is a hotly debated one, with publications supporting multiple causal hypotheses, we do not think that (positive) publication bias has a major impact on our findings, although we cannot formally exclude this. We did not search all of the available literature databases, although it is unlikely they should have yielded important studies unlisted in Medline (as illustrated by our lack of additional findings in PsycINFO).

## Conclusions and Future Perspectives

Despite our clear initial research question, this systematic review did not provide a single answer to the question of how depressive symptoms and later dementia are related. According to our review effort, grouping multiple large studies that provide conflicting results, it remains unclear whether depressive symptoms in the elderly are a risk factor for or a prodromal symptom of dementia. They still may be related mainly through common causal factors, e.g., aging or vascular disease. It seems unlikely that they are not at all related, or only in an indirect way–for example, evidence does not support the hypothesis that a negative emotional response to incipient cognitive symptoms alone can explain the connection between depressive symptoms and dementia.

Several interesting issues raised in some of the studies included, although outside the scope of this review, also deserve further evaluation. These include but are not limited to the role of gender and genetic factors, systemic inflammation and cerebrovascular disease, different etiologies of dementia developed (utilizing recent advances in pathological classification), the nature and severity of symptoms, in modulating the odds of developing dementia in depressive elders.

We deduce from this systematic review that depressive symptoms can be an independent risk factor for as well as a prodromal manifestation of dementia. In some cases, they may both stem from common risk factors such as cerebrovascular disease. In others, they may not have causal connections at all and simply occur together by chance—as two separate yet prevalent neuropsychiatric diseases with overlapping and prevalent risk factors. It remains, on the basis of these findings, challenging to identify those depressed elders at an increased risk of later dementia in clinical practice and, by extension, who would benefit from specific interventions to attenuate this risk.

Therefore, further research is needed to unravel the association between depression and dementia. Preferentially, this research should use a large database to have sufficient statistical power to determine which risk factors–possibly a combination of clinical characteristics and biomarkers, hardly available at all in the studies we examined—increase the risk of conversion to dementia in the depressed elderly. These risk factors should subsequently be validated in prospective, longitudinal clinical studies including elders with and without depressive symptoms in whom clinical, biochemical, and neuropsychological follow-up will decipher which (sub)group later develops cognitive deterioration and dementia. These risk factors can then be incorporated into a clinically useful risk score, of paramount importance for future efforts in the prevention of dementia—and therefore of interest to clinicians, researchers, and patients worldwide.

## Author Contributions

WW, CB, and SE conceived the idea for this manuscript. WW performed the database searches and wrote the first drafts. CB and SE critically reviewed and commented on these drafts. All authors contributed to manuscript revision. All authors read and approved the submitted version.

## Funding

This work was supported by the Geneeskundige Stichting Koningin Elisabeth/Fondation Médicale Reine Elisabeth. WW is a PhD fellow of the Research Foundation Flanders (FWO-Vlaanderen, grant no. 11E8620N).

## Conflict of Interest

The authors declare that the research was conducted in the absence of any commercial or financial relationships that could be construed as a potential conflict of interest.
