# Supplementary material for: Depressive Symptoms in the Elderly—An Early Symptom of Dementia? A Systematic Review
Source: Front Pharmacol. 2020 Feb 7;11:34. doi: 10.3389/fphar.2020.00034 (PMC7020568; doi:10.3389/fphar.2020.00034)
Supplement: Supplementary file 1 [file DataSheet_1.docx]

Supplementary Material

**Supplementary table 1: Search terms identified:**

| P | I | C | O |
| --- | --- | --- | --- |
| **Adults 65 or older with major depressive disorder and no dementia** | **Diagnostic evaluation of neurodegeneration: MRI, PET, neurochemistry** | **Cognitively healthy older adults without depression** | **(An increased) risk of dementia later in life** |
| Depression (includes variant wordings such as depressive, MDD,… in MeSH) | MRI | x | Incidence |
| Dementia | Magnetic Resonance Imaging |  | Risk |
| Older adults, elderly, 65 | PET |  | Hazard |
|  | Positron-emission tomography |  | Cohort studies |
|  | Biomarkers |  | Diagnosis |
|  | Cerebrospinal fluid |  |  |
|  | CSF |  |  |
|  | Tau |  |  |
|  | Amyloid |  |  |

**Example searches and results for Medline:**

Depression AND Dementia AND Biomarkers 356, + risk 136, + incidence 66, + hazard 6, + cohort 79

Depression AND Alzheimer AND Biomarkers 219, + risk 66, + incidence 39, + hazard 4, + cohort 49

Depression AND Dementia AND cerebrospinal fluid 259, + risk 45, + incidence 26, + hazard 1, + cohort 49

Depression AND Alzheimer AND cerebrospinal fluid 165, + risk 24, + incidence 14, + hazard 1, + cohort 31

Depression AND Dementia AND Amyloid 444, + risk 136, + incidence 60, + hazard 2, + cohort 61

Depression AND Alzheimer AND Amyloid 406, + risk 106, + incidence 48, + hazard 1, + cohort 44

Depression AND Dementia AND Tau 222, + risk 55,  + incidence 32, + hazard 1, + cohort 48

Depression AND Alzheimer AND Tau 155, + risk 35, + incidence 20, + hazard 0, + cohort 31

Depression AND Dementia AND MRI 356, + risk 192, + incidence 135, + hazard 10, + cohort 203

Depression AND Alzheimer AND MRI 269, + risk 62, + incidence 51, + hazard 2, + cohort 78

Depression AND Dementia AND Positron Emission Tomography 163, + risk 44 , + incidence 23, + hazard 7, + cohort 32

Depression AND Alzheimer AND Positron Emission Tomography 87, + risk 20 , + incidence 14, + hazard 1,  + cohort 18

Depression AND Dementia AND neuropsychological test 3757, + risk 892, + incidence 1113, + hazard 52, + cohort 1033

Depression AND Alzheimer AND neuropsychological test 1586, + risk 331, + incidence 422, + hazard 19, + cohort 43

| Study | Representativeness | Selection | Exposure | Outcome at baseline | Comparability | Assessment | Follow-up length | Follow-up quality | Comment |
| --- | --- | --- | --- | --- | --- | --- | --- | --- | --- |
| Brodaty 2012 | * | * | * | * | ** | * | - | * | Short follow-up. Interview by phone, sometimes informant. Only link for depression, not other psychological symptoms |
| Burke 2016 | * | * | * | * | ** | * | * | * | Very large and long study. Several types of depression. Increasing risk when corrected for all kinds of other factors. |
| Devanand 1996 | - | * | * | * | ** | * | - | * | Relatively short follow-up. |
| Ezzati 2019 | * | * | * | * | ** | * | * | * | Modern and well worked out study with many sub-analyses of symptoms and effects alike. |
| Irie 2008 | - | * | * | * | ** | * | * | * | Only men. Interesting link with APOEe4. Multiple corrections. |
| Saczinsky 2010 | * | * | * | * | ** | * | * | * | Long follow-up, multiple corrections, correlation with severity of symptoms, exclude MCI (so no reverse causality). |
| Spira 2012 | - | * | * | * | ** | * | * | * | Only very old women. Few corrections that do support the connection. |
| Becker 2009 | * | * | * | * | ** | * | * | * | Small cohort. Negative for APOE, education,… - underpowered? |
| Blasko 2010 | * | * | * | * | - | * | * | * | Somewhat small, never depressed cohort. No effect of APOE, gender, stroke,…? |
| Geerlings 2008 | * | * | * | * | ** | * | * | - | Wide CI. Younger participants. Only 33 cases of dementia. Power? |
| Kim 2010 | * | * | * | * | * | * | - | - | Self-report. There is a link with APOEe4. Connection through disability & activity, sex? Not vascular risk. Short follow-up. |
| Lindsay 2002 | * | * | - | * | ** | * | * | * | Only AD. Protective effect of NSAIDs, no effect of family history, trauma,.. (?), long questionnaires. |
| Mossaheb 2012 | * | * | * | * | - | * | * | * | Only loss of interest (~apathy?) |
| Palsson 1999 | * | * | * | * | - | - | * | * | Small cohort. Only MMSE as cognitive test. Risk for earlier depression. |
| Vinkers 2004 | * | * | * | * | * | * | * | * | Argue for reverse causation/psychological reaction. |
| Almeida 2017 | * | * | * | * | * | * | * | * | Long follow-up, large cohort. Some risk and prodrome? Healthcare records not optimal, multiple corrections. |
| Chen 1999 | * | * | * | * | * | * | * | * | Wide CI for risk so not excluded. Argue for early symptom. |
| Fuhrer 2003 | * | * | * | * | ** | * | * | * | Only in men, although different cut-offs. Vascular hypothesis prevails. MRI. |
| Gatz 2005 | * | * | * | * | ** | * | * | * | Don’t exclude risk as additional role |
| Geerlings 2000 | * | * | * | * | ** | * | * | * | Mainly in those with higher education. Two studies. |
| Lenoir 2011 | * | * | * | * | ** | * | * | * | Large cohort, multiple corrections, MRI data. No effect for earlier episodes. |
| Li 2011 | * | * | * | * | ** | * | * | * | Large study, long follow-up. |
| Mirza 2016 | * | * | * | * | ** | * | * | * | Corrections for classic factors. Evaluated different trajectories of symptoms. |
| Palmer 2007 | - | * | * | * | ** | * | * | * | Very small cohort, but good differentiation from MCI cases. |
| Verdelho 2013 | - | * | * | * | ** | * | - | * | Had to have white matter lesions – many Vascular dementia cases. |
| Ganguli 2006 | * | * | * | * | * | * | * | * | Only cross-sectional correlation, none in interaction models. Cognitive symptoms of depression? |
| Kaup 2016 | * | * | * | - | * | - | * | * | Records or prescription-based diagnosis of dementia not optimal. |
| Luppa 2013 | * | * | * | * | ** | * | * | * | Multivariate analyses, corrections made the correlation disappear. |
| Wilson studies | - | * | * | * | * | * | * | * | See discussion. |

Supplementary table 2: Newcastle-Ottawa Scale (NOS) based design bias assessment of studies - www.ohri.ca/programs/clinical_epidemiology/oxford.asp. Stars are allocated when certain predefined characteristics in study design are met. Two stars can be obtained in the comparability section (element 5).

| Study | Category | Rationale for primary classification in hypothesis categories |
| --- | --- | --- |
| Brodaty 2012 (26) | A1 – Risk Factor | Results: “*Depression predicted dementia at follow-up (OR 3.67 95% CI 1.1–12.5, p = 0.038) but not MCI (OR 0.87 95% CI 0.5–1.5, p = 0.63)*.” Discussion: “*Overall NPS did not predict*  *decline for either baseline NCI or MCI groups (…) our results showed an association between*  *baseline depression and incident dementia, which is consistent with some studies examining conversion from MCI or NCI, but not all.”* No difference after corrections for age, sex, NPI score, education. |
| Burke 2016 (27) | A1 – Risk Factor | Several types of depression, including past episodes, all increase risk. Increasing risk when corrected for all kinds of other factors, which is an argument against reverse causation. Very long follow-up (relative argument against prodrome) and large cohort. Combination with APOE-genotypes suggest dual-hit/gene-environment model. Authors do not contradict the prodromal hypothesis, but mainly list depression as one of several risk factors. |
| Devanand 1996 (28) | A1 – Risk Factor | *“Effect of baseline depressed mood on the end-point diagnosis of dementia was evaluated in a Cox model. Depressed mood at baseline was associated with an increased risk of incident dementia (RR, 2.94; 95% CI, 1.76 - 4.91; P<.001). This effect remained after adjustment for age,*  *gender, education, language of assessment, BIMC and BFAS scores (RR 2.05;*  *95% CI 1.16 - 3.62; P<.02). Similar results were obtained when the total Hamilton Rating Scale*  *for Depression score was used as the depression variable, with the use of the same covariates (RR 1.07 per point)”…* The authors note that this risk may stem from prodromal manifestations of AD or dementia, but *“… in the longitudinal data set, when the Cox analyses were conducted after removal of the subgroup with cognitive impairment at baseline evaluation, depressed mood*  *remained a risk factor for dementia. This argues against the notion that depressed mood is more a prodromal or early sign than a risk factor for dementia.* Further argue against the cognitive-reserve or emotional response possibilities in their discussion section. |
| Ezzati 2019 (23) | A1 – Risk Factor | GDS-per point analysis is interesting, as is correction for BIMC, and major depressive episodes. These add to their strength in defending a true ‘risk’ hypothesis, rather than a common causal model or reserve causation. Effect is clearer AFTER 3 years so risk > prodrome. Clear stance by authors on this point. |
| Irie 2008 (29) | A1 – Risk Factor | Risk elevation that is multiplied by genotype – lending support to an additional risk model/double-hit hypothesis. Still significant after corrections for various factors. Correction for baseline cognition and emotional symptoms – use this as an argument against pure prodromal possibility. They discuss the possibility of vascular factors mediating this risk, but conclude that they cannot make any new statements on the matter (although their corrections for these factors did not seem to influence findings in a major way). N.B. only men. |
| Saczinsky 2010 (30) | A1 – Risk Factor | Do not formally exclude other possibilities but very long follow-up (>5 and up to 17y) and excluding MCI cases argues against (some cases of) reverse causation/prodromal symptoms. Multiple corrections for other factors (e.g. vascular) did not affect results. Limitations are clearly discussed. |
| Spira 2012 (31) | A1 – Risk Factor | Oldest old females only with high fitness (no nursing homes). No major differences between subgroups at baseline. Authors argue mainly for the (true) risk hypothesis in their discussion; including the observation that severity of symptoms is linked with poorer cognitive performance in the future. |
| Becker 2009 (40) | A2 – Not a risk factor | Only cross-sectional association – cognitive reserve hypothesis? Did not find any other risk factors apart from age – power issue? |
| Blasko 2010 (41) | A2 – Not a risk factor | Some associations between plasma Ab42 and depressive symptoms, as well as future AD. No risk relationship between GDS and later dementia (nor APOE, stroke,…) |
| Geerlings 2008 (42) | A2 – Not a risk factor | Wide confidence interval concerning late onset depression, resulting in a lack of significance. Relatively young participants. Only 33 cases of dementia. Power? N.B. increased risk for EARLY onset (major) depression, so doesn’t definitely contradict a correlation. |
| Kim 2010 (32) | A2 – Not a risk factor (in general) | Only significant (yet highly so!) after being adjusted in the presence of ‘susceptible’ APOE-genotypes. Cfr. Irie (29). |
| Lindsay 2002 (43) | A2 – Not a risk factor | Very broad analysis of all kinds of epidemiological factors on AD risk (e.g. coffee and wine consumption, exercise, history of depression,…). No correlation found with depression. |
| Mossaheb 2012 (44) | A2 – Not a risk factor | Authors find no link and argue that depressive symptoms in elders are mainly due to “genuine” depression. They did find a link with loss-of-interest-symptoms of depression (which may represent apathy, a known prodromal factor of neurodegenerative disorders? Could therefore also be included under category B when including loss of interest under depressive symptoms) |
| Palsson 1999 (45) | A2 – Not a risk factor | Small and somewhat dated study using limited resources that was negative concerning our question. Did, however, find a risk association with early onset depressive disorders. |
| Vinkers 2004 (46) | A2 – Not a risk factor | The authors find no increased future risk but note that already cognitively impaired people often develop depressive symptoms – i.e. depressive symptoms are a common symptom of dementia (not prodromal in the strict sense, since cognitive decline is already measurable). |
| Almeida 2017 (47) | B – Prodromal symptom | Symptom trajectories and findings only during the first five years lead the authors to defending a mainly prodromal position. Correlation with severity might support a risk role, but no effect of antidepressants is advanced as a counter-argument. |
| Chen 1999 (48) | B – Prodromal symptom | No (significant) risk of dementia or AD following depressive symptoms. Strong risk of developing depressive symptoms after the onset of cognitive disorders -> argue for reverse causation. |
| Fuhrer 2003 (49) | B – Prodromal symptom | Risk elevation only significant in men (N.B. differing cut-off values for depressive symptoms based on gender!). The authors find much to say for a ‘vascular depression’ model that causes both mood symptoms and cognitive impairment, and therefore posit a prodromal position. No effect of ‘distant’ (i.e. early) depression. |
| Gatz 2005 (33) | B – Prodromal symptom | Although an element of true risk is allowed for, the authors argue that because neither past depression nor the duration/severity of symptoms impact conversion, as well as the fact that this occurs during the first few years after screening, a prodromal model better fits their findings. |
| Geerlings 2000 (50) | B – Prodromal symptom | Combination of two cohort studies. The authors only found an increased risk among highly educated subjects, more pronounced in severe cases. Through the cognitive reserve paradigm, they take this to be a sign of early pathological changes. It is unclear how they refute the true risk hypothesis, which may still hold true. |
| Lenoir 2011 (51) | B – Prodromal symptom | Since no effect was observed for earlier episodes and major depressive disorder, as well as a negative result for AD in contrast to a highly increased chance of Vascular dementia (with severe and/or increasing symptoms), the authors conclude that depressive symptoms are part of the symptom spectrum of cerebrovascular/-degenerative disease rather than a true risk factor. |
| Li 2011 (52) | B – Prodromal symptom | Authors prefer the prodromal hypothesis because of a lack of effect for self-reported earlier episodes as opposed to recent symptoms. Some alternative options, however, are discussed at length and left open. |
| Mirza 2016 (53) | B – Prodromal symptom | Stances taken in this article are mainly based upon the authors (relatively unique) assessment of symptom trajectories over time. Since only the ‘severe and increasing’ trajectory was associated with the development of dementia, they suggest that these may represent the emergence of degenerative disease (when correcting for several other factors). |
| Palmer 2007 (54) | B – Prodromal symptom | Differentiation from cases developing from MCI and from normal cognitive status. Several arguments are advanced for the early-symptom-model by pointing to pathological studies, although the possibility of a true risk factor is not explicitly addressed or refuted. |
| Verdelho 2013 (55) | B – Prodromal symptom | Much like Lenoir (51), conclusions are drawn from striking additive/synergistic effects with (vascular) white matter lesions, leading to authors to *suspect “subtle ongoing organic dysfunction”* as the cause of depressive symptoms. |
| Ganguli 2006 (59) | D – Neither/something else | Since only cross-sectional and no long term interactions were found, the authors suggest that cognitive symptoms may be symptoms of depression. (Similar cohort to (48) in category B) |
| Kaup 2016 (60) | C – Possibly prodrome and risk | Given an especially clear link between high/increasing symptom burden and later dementia, both true risk and prodromal paradigms are substantiated by the authors. |
| Luppa 2013 (61) | D – Neither/something else | Although some correlations were found in exploratory analyses, all disappeared in multivariate regression models. |
| Wilson studies (20, 21, 22) | D - Something else (entirely) | See discussion section, interesting findings that are challenging to link with this or that specific hypothesis category we created. |

Supplementary table 3
